# Blood Biomarkers Discriminate Cerebral Amyloid Status and Cognitive Diagnosis when Collected with ACD-A Anticoagulant

**DOI:** 10.2174/0115672050271523231111192725

**Published:** 2023

**Authors:** Zachary D. Green, Paul J. Kueck, Casey S. John, Jeffrey M. Burns, Jill K. Morris

**Affiliations:** 1 Alzheimer’s Disease Research Center, University of Kansas, Kansas City, KS, 66160, United States; 2 Department of Neurology, University of Kansas Medical Center, Kansas City, KS, 66160, United States

**Keywords:** Alzheimer’s disease, biomarkers, amyloid, plasma, processing, ACD, anticoagulant

## Abstract

**Background:**

The development of biomarkers that are easy to collect, process, and store is a major goal of research on current Alzheimer’s Disease (AD) and underlies the growing interest in plasma biomarkers. Biomarkers with these qualities will improve diagnosis and allow for better monitoring of therapeutic interventions. However, blood collection strategies have historically differed between studies. We examined the ability of various ultrasensitive plasma biomarkers to predict cerebral amyloid status in cognitively unimpaired individuals when collected using acid citrate dextrose (ACD). We then examined the ability of these biomarkers to predict cognitive impairment independent of amyloid status.

**Methods:**

Using a cross-sectional study design, we measured amyloid beta 42/40 ratio, pTau-181, neurofilament-light, and glial fibrillary acidic protein using the Quanterix Simoa^®^ HD-X platform. To evaluate the discriminative accuracy of these biomarkers in determining cerebral amyloid status, we used both banked plasma and 18F-AV45 PET cerebral amyloid neuroimaging data from 140 cognitively unimpaired participants. We further examined their ability to discriminate cognitive status by leveraging data from 42 cognitively impaired older adults. This study is the first, as per our knowledge, to examine these specific tests using plasma collected using acid citrate dextrose (ACD), as well as the relationship with amyloid PET status.

**Results:**

Plasma AB42/40 had the highest AUC (0.833, 95% C.I. 0.767–0.899) at a cut-point of 0.0706 for discriminating between the two cerebral amyloid groups (sensitivity 76%, specificity 78.5%). Plasma NFL at a cut-point of 20.58pg/mL had the highest AUC (0.908, 95% CI 0.851–0.966) for discriminating cognitive impairment (sensitivity 84.8%, specificity 89.9%). The addition of age and apolipoprotein e4 status did not improve the discriminative accuracy of these biomarkers.

**Conclusion:**

Our results suggest that the Aβ42/40 ratio is useful in discriminating clinician-rated elevated cerebral amyloid status and that NFL is useful for discriminating cognitive impairment status. These findings reinforce the growing body of evidence regarding the general utility of these biomarkers and extend their utility to plasma collected in a non-traditional anticoagulant.

## 1. INTRODUCTION

Alzheimer’s Disease (AD) is the most common form of dementia, but there is no cure and few treatment options. Better classification of clinical impairment is a key step towards improving clinical trial design. In recent years, the “A/T/N” framework was developed to classify amyloid, tau, and neurodegeneration in participants [1] and can be used as a tool to identify groups that may benefit from various pharmacological interventions. Gold standard A/T/N measures are performed using positron emission tomography (PET) imaging and cerebrospinal fluid [2–4]. However, these are expensive and present as a deterrent for recruitment [4], underscoring the need for blood-based biomarkers.

Blood-based biomarkers have quickly developed in recent years. For instance, the plasma ratio of AB42:AB40 discriminates between individuals with elevated cerebral amyloid and controls [5–7]. This ratio is outperformed by other biomarkers (such as neurofilament light chain (NfL) or phospho-tau 217 (pTau217) for discriminating between cognitive impairment due to AD and other causes of dementia [8]. However, direct comparisons between the high-sensitivity platforms and methods used in AD blood biomarker research demonstrate some differences in performance and reliability [9]. While plasma amyloid tests, such as Quanterix Simoa, accurately discriminate between individuals with and without cerebral amyloid [10–13], the performance varies between the studies [9, 14].

Importantly, pre-analytical factors, such as collection tube type, are an important consideration when examining biomarker results [15, 16], as certain research questions may require specific anticoagulant types. For instance, acid citrate dextrose solution A (ACD-A) improves outcomes for studies focused on microparticles, such as extracellular vesicles (EVs), without interfering with downstream protein or RNA analyses [17]. Platelet removal is particularly important when extracellular vesicles are the target of research [18], and in platelet-free plasma, ACD-A inhibits *ex vivo* microparticle generation [19]. ACD-A is superior to EDTA for the preparation of platelet-rich plasma (PRP) [20], showing better preservation of platelet morphology, physiological growth factor concentration [21], and intraplatelet signaling [22]. Blood collected with ACD-A has also been historically used in studies on mitochondrial function, as EDTA is a calcium chelator and may interfere with such functional outcomes and the generation of cell lines for these analyses [17, 23, 24]. For studies focusing on platelet physiology, EV-related functions, or mitochondrial health and AD, the acquisition of ACD-A plasma has historically, and often exclusively, been banked. This study seeks to validate the measurement of emerging AD biomarkers in ACD-A plasma, allowing for the simultaneous assessment of such functional measures and AD biomarkers using a single blood sample and in previously banked samples for which no EDTA sample was collected. To our knowledge, no studies have quantified emerging AD biomarkers in ACD-A plasma. Our goal was to evaluate the performance of ACD-A plasma biomarkers in determining AD risk (elevated vs. non-elevated cerebral amyloid) and diagnosis (cognitively healthy; CH vs. cognitively impaired). For those in which it was available, we specifically evaluated the performance of such biomarker assays in ACD-A plasma.

## 2. METHODS

### 2.1. Participant Characteristics

For this project, we cross-sectionally evaluated biomarkers in 183 participants (140 cognitively healthy, CH older adults and 43 cognitively impaired participants) with banked ACD-A plasma. All participants were enrolled in the KU Alzheimer’s Disease Center Registry (IRB #11132) or the Alzheimer’s Prevention through Exercise (APEx) study (IRB #13376). In addition, 18F AV45 data was available for all CH participants but not for cognitively impaired participants. One individual was excluded from analyses based on having an extreme outlier biomarker value (>3 standard deviations from mean), resulting in a cognitively impaired cohort of 42 individuals. The KUMC IRB approved the protocol, and written informed consent was obtained from all participants.

All participants were assessed by a trained clinician using the Alzheimer’s Disease Centers’ Uniform Data Set neuropsychological test battery 2.0 (UDS 2.0) [25], Mini-Mental State Examination, and the Clinical Dementia Rating (CDR) [26]. These clinical and cognitive assessments were reviewed at consensus diagnosis conferences to determine eligibility. All CH older adults were CDR 0 with no clinically significant cognitive deficits. Cognitively impaired participants had a global CDR of 0.5 or higher. Clinical and cognitive data were reviewed at a consensus diagnostic conference attended by ADC clinicians, neuropsychologists, and nurses. The primary etiology for all cognitively impaired participants in this study was probable AD.

### 2.2. Plasma Collection and Analysis

Upon arrival at the KU Clinical and Translational Science Unit following an overnight fast, height and total body mass were measured. Blood was collected using ACD-A serum vacutainer tubes. All blood processing steps, including centrifugation, were performed at room temperature. ACD tubes were spun at 1200 × g for 12 minutes (acceleration 9, brake 0). Plasma was aliquoted into cryotubes for storage at −80C until analysis. For serum collection, red top tubes were centrifuged at 1200 × g for 12 minutes (acceleration 9, deceleration 0) prior to storage at −80C.

All biomarkers were measured in ACD-A plasma using a Quanterix Simoa^®^ HD-X except for GFAP, which was measured in serum due to sample volume constraints. Serum samples were only available for the CH groups, so GFAP analyses were limited to the CH groups. AB42 and AB40 were measured with the Neurology 3-Plex A (N3PA) assay. Our decision to use the N3PA kit, as opposed to the Neurology 4-plex kit, was informed by comparisons between the kits made on a separate developmental cohort (Figs. S1 and S2). Singleplex assays were used to measure pTtau181 and NfL. Glial fibrillary acidic protein (GFAP) was measured using the Neurology 2-Plex B (N2PB). All analyses were performed as per the manufacturer’s instructions. As the N3PA kit is only validated for EDTA plasma, we performed partial validation of the N3PA kit in ACD-A plasma, according to Andreasson *et al.* [27]. This partial validation consisted of measuring assay sensitivity, parallelism, repeatability, and intermediate precision. The performance of the N3PA kit in ACD-A plasma succeeded (Table S1). Plasma total tau showed poor correspondence between the tube types (R2 = 0.32, Fig. S1) and is known to have low diagnostic utility and poor correspondence between CSF and plasma [28, 29] and was not included here. We did not carry out a partial validation for NfL or p-tau-181 due to sample and resource constraints. However, as the N2PB assay used to measure GFAP was performed in serum (due to the same constraints), we had N2PB-NfL serum measurements to compare with the single-plex-NfL ACD-A measurements. The correspondence between these measurements was high (R2 = 0.79, Fig. S3). Due to this correspondence, we did not include serum N2PB-NfL measurement in our ROC analysis.

### 2.3. Assessment of Cerebral Amyloid

18F-AV45 PET images were obtained on a GE Discovery ST-16-PET/computed tomography scanner after intravenous administration of 370 MBq of flobetapir F-18. Two PET frames (5 min duration, ~50 min post-injection) were averaged and attenuation corrected. The resulting image was normalized to the cerebellum to generate standard uptake value ratios (SUVR) using MIMneuro [30, 31]. Quantitative SUVR information was generated for 6 ROIs (anterior cingulate, posterior cingulate, precuneus, inferior medial frontal, lateral temporal, and superior parietal cortex). A global SUVR was produced by summing these ROIs.

For classifying individuals as “elevated” or “not elevated” based on PET results, we used a “VisQ Read” described previously and showed an increase in inter-rater reliability 30. Briefly, both a “Visual Read” and a “Quantitative Read” were performed. Raters were then asked to consider the cortical projections, “Visual Read”, and “Quantitative Read”, and provide a final interpretation, or “VisQ Read”, of each scan as “elevated” or “non-elevated”. Individuals were considered to be elevated when two or more raters agreed that the “VisQ Read” was “elevated”.

### 2.4. Statistical Analysis

Statistics were performed in SPSS v26. We examined statistically significant differences in demographic information using independent samples t-tests for continuous variables and chi-squared tests for categorical information. Two linear regressions were conducted for each plasma biomarker: one testing for differences between the non-elevated and elevated groups and one testing for differences between the pooled cognitively unimpaired groups and the cognitively impaired group. Each regression was additionally conducted with age, sex, and *APOE4* entered as covariates to examine if significant effects remained. Pearson’s correlation coefficient was used to examine the association between individual biomarkers and between the plasma biomarkers and amyloid-PET results. For the ROC analysis, cut points were determined using Youden’s index, which equally weights sensitivity and specificity when evaluating test performance. ROC curves were constructed for the available plasma biomarkers, examining their ability to discriminate both amyloid status and cognitive impairment.

## 3. RESULTS

### 3.1. Participant Characteristics

Cohort characteristics are described in Table 1. Of the 182 participants included, there were 65 CH individuals with low cerebral amyloid (“non-elevated”), 75 CH with high cerebral amyloid (“elevated”), and 42 cognitively impaired individuals with a primary etiology of probable AD. The mean age was 71.3 ± 5.3 for the non-elevated group, 72.2 ± 5.0 for the elevated group, and 73.5 ± 9.4 for the cognitively impaired group. There was not a significant difference in age between amyloid groups (*p*=0.292), although there was between CH and impaired (*p*<.001). Sex did not differ between the amyloid group (*p*=0.528) or impairment groups (*p*=.618). Education did not differ between the non-elevated and elevated groups (*p*=0.830) but was significantly lower in the cognitively impaired group when compared to the combined CH (*p*=0.004).

There was a higher proportion of *APOE4* carriers in the elevated than in the non-elevated group (*p*<0.001). There was also a higher proportion of carriers in the cognitively impaired group compared to the combined CH groups (*p*=.013). Further testing between the individual groups demonstrated a significant difference between the impaired group and the non-elevated amyloid group alone (*p*<0.001) but not between the impaired group and the elevated amyloid group alone (*p*=0.357). MMSE did not differ between the non-elevated and elevated groups (*p*=0.763) but was significantly lower in the cognitively impaired group when compared to the CH groups (*p*<0.001).

### 3.2. Plasma Biomarkers, Specifically AB42/40 Ratio, Predict Elevated Cerebral Amyloid Status

**Fig. (1).**
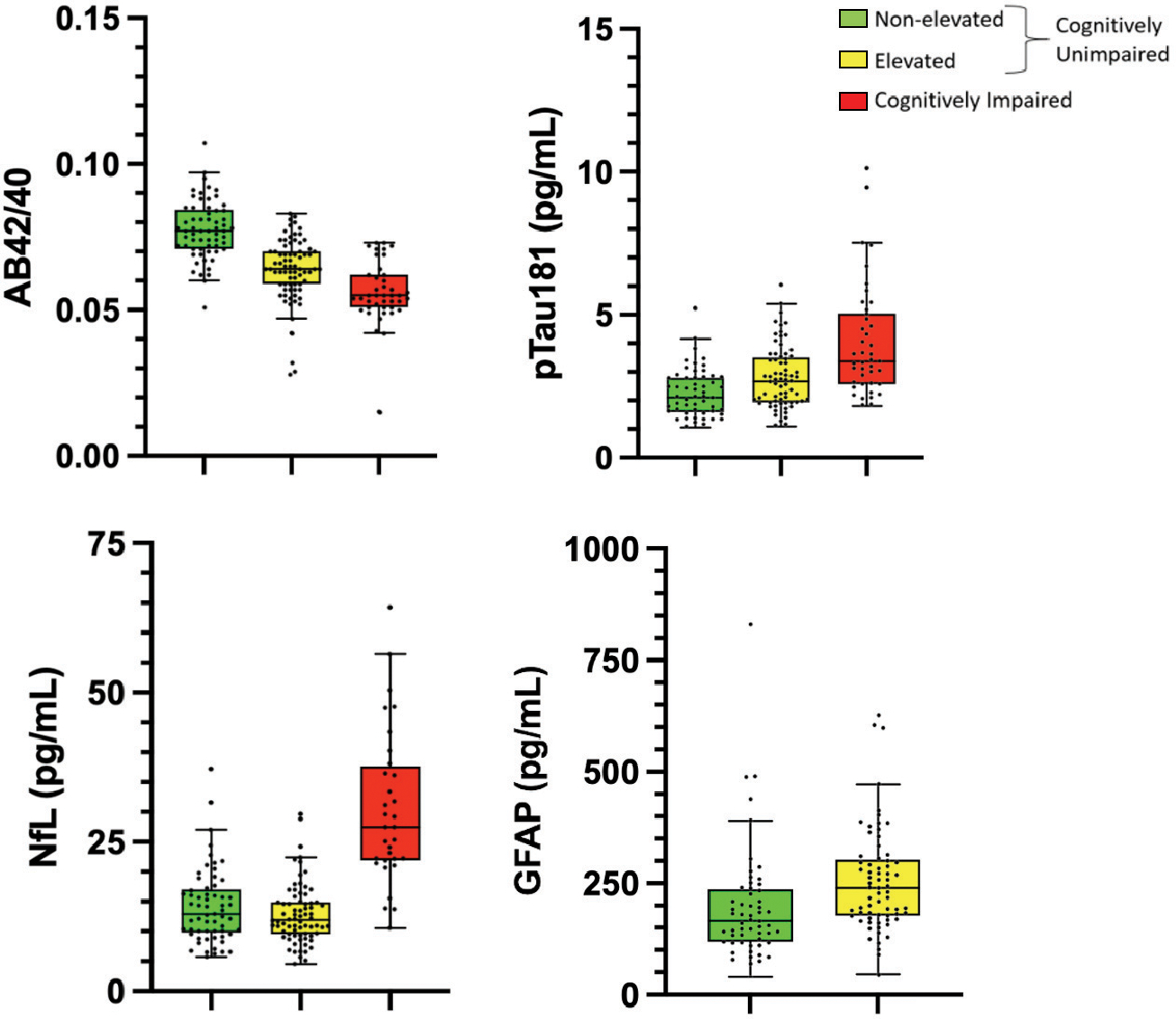
ACD plasma A/T/N biomarkers differ between groups. Plots of individual biomarker values and group mean for each plasma biomarker showing how ACD plasma A/T/N biomarkers differ between groups. Bars represent the 25th percentile, mean, and 75^th^ percentile. Whiskers correspond to the 25^th^ percentile and 75^th^ percentiles minus and plus (respectively) the interquartile range. **Abbreviations:** AB, amyloid beta; pTau, phosphorylated tau; NfL, neurofilament-light; GFAP, glial fibrillary acidic protein.

The mean, standard deviation, and significance of the effect of amyloid status on each plasma biomarker and amyloid-PET signal are summarized in Table 1 and visualized in Fig. (1). Regression showed an effect of elevated status on plasma AB42, with the non-elevated group (11.49 ± 2.36) showing higher levels of AB42 than the elevated (8.93 ± 2.30) amyloid group. There was no significant effect of elevated status on plasma AB40 (non-elevated, 150.3 ± 33.4; elevated, 140.5 ± 30.5). The AB42/40 ratio was significantly lower in the elevated group (.0638 ± .0110) when compared to the non-elevated group (.0773 ± .0099), corresponding to a mean decrease of 17.5%. Of the non-amyloid plasma markers, GFAP (non-elevated, 200.7 ± 131.7; elevated, 255.2 ± 115.1) and pTau181 (non-elevated, 2.25 ± 0.84; elevated, 2.80 ± 1.09) differed significantly based on cerebral amyloid status, while NfL did not (non-elevated, 14.05 ± 6.15; elevated, 12.88 ± 5.03). The effect of group amyloid status on each biomarker was not affected by the inclusion of covariates (age, sex, and *APOE4* carrier status).

**Fig. (2).**
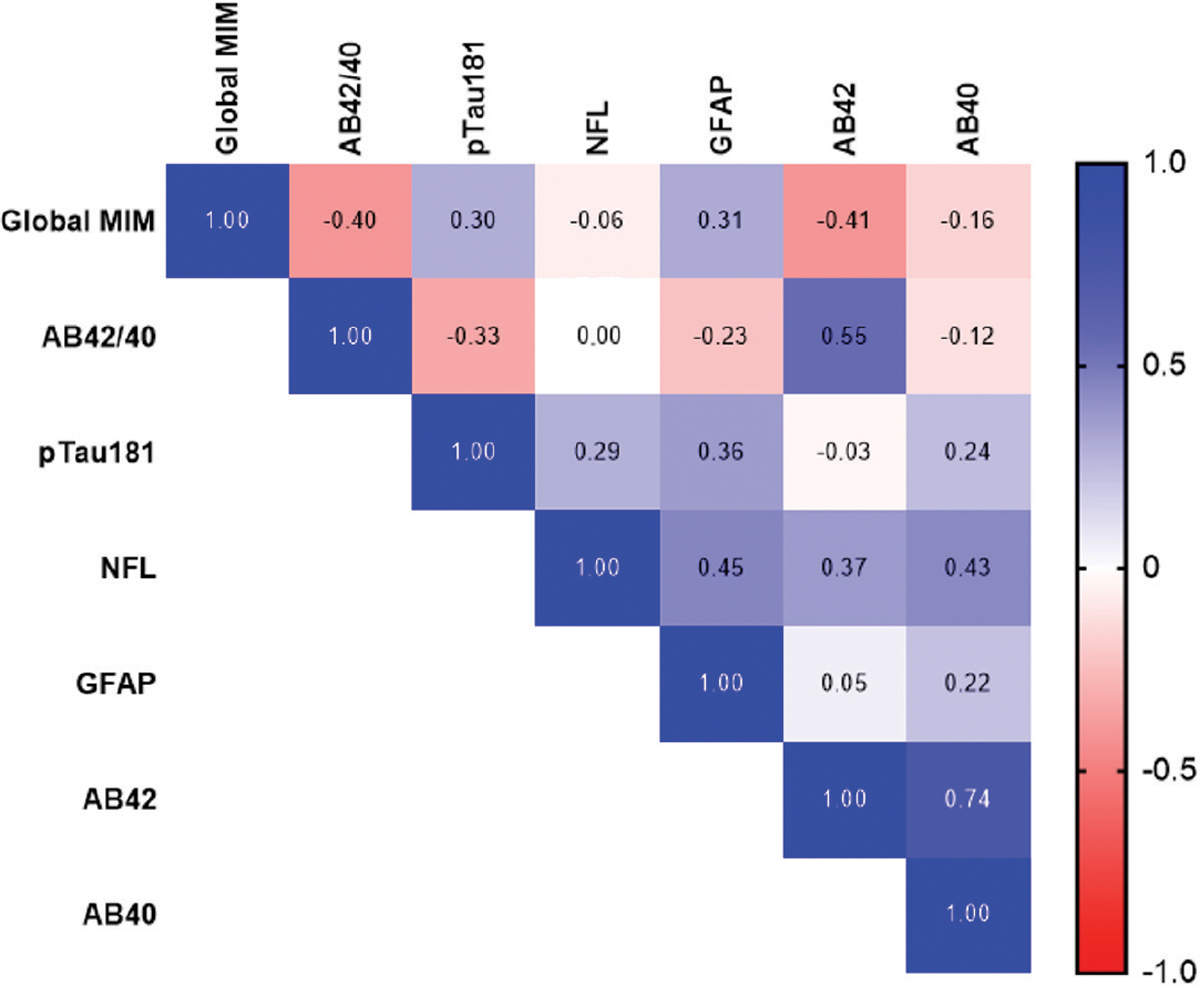
Correlations between amyloid-PET and plasma biomarkers (cognitively unimpaired only) Pearson’s correlation heat maps for the biomarkers measured in the cognitively unimpaired group. **Abbreviations:** AB, amyloid beta; pTau, phosphorylated tau; NFL, neurofilament-light; GFAP, glial fibrillary acidic protein.

The Global MIM signal in each group strongly reflected the clinician rating (non-elevated, 1.015 ± .048; elevated, 1.288 ± .160). This group difference held for each individual ROI evaluated (Table S2), though the effect was strongest in the precuneus (non-elevated, 1.042 ± .068; elevated, 1.38 ± .207). Correlations between Global MIM and individual biomarkers are shown in Fig. (2). Plasma AB42/40 ratio (r=−.402, *p*<0.001) and AB42 (r=−0.41, *p*<0.001) are the most strongly associated with the Global MIM signal. Correlations between individual biomarkers in the entire cohort, as well as in the impaired group only, can be found in Table S3 and Table S4, respectively.

**Fig. (3).**
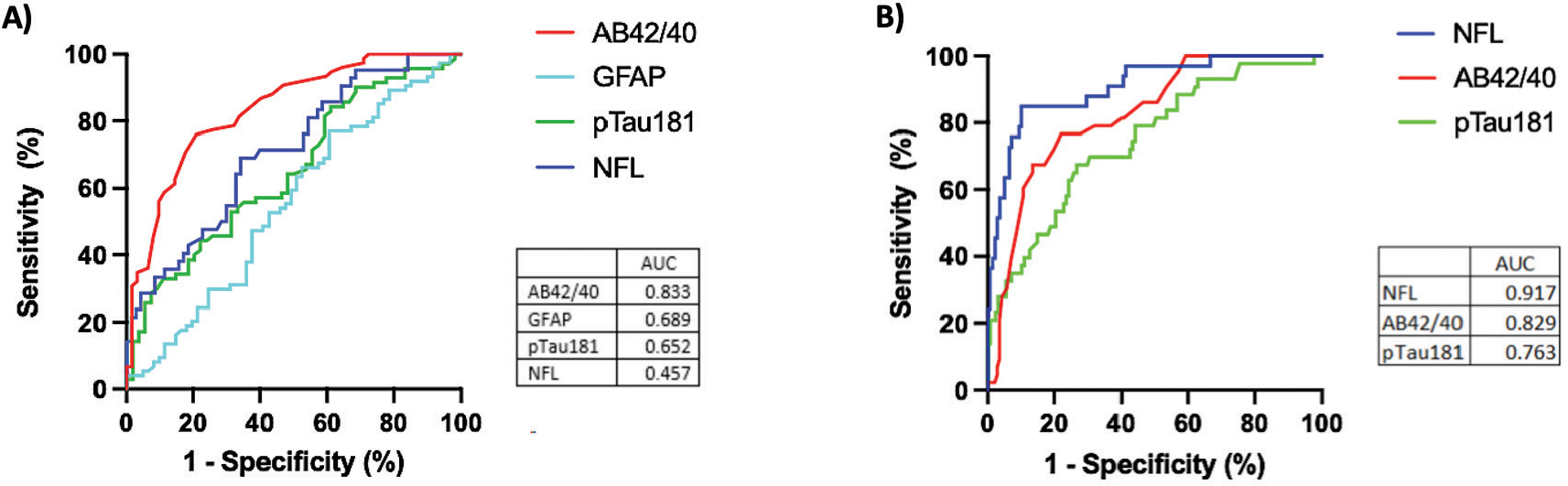
Performance of ACD-plasma biomarkers for classifying elevated amyloid status and cognitive diagnosis. Performance of ACD-plasma biomarkers for classifying elevated amyloid status in cognitively unimpaired older adults. The ROC curves show each plasma biomarker’s ability to discriminate (**A**) cerebral amyloid status (cognitively unimpaired cohort only) and (**B**) cognitively impaired status. The AUC value for each curve is listed in the bottom right of the plots. Plasma GFAP was not included in the analysis for diagnosis, as it was not available in the cognitively impaired subcohort.

AB42/40 had the highest AUC (0.833, 95% C.I. 0.767–0.899) for the ROC analysis in discriminating between the two cerebral amyloid groups (Figs. 3A and B). Using Youden’s Index, the optimal cut-point was determined to be 0.0706, which resulted in a sensitivity of 76% and a specificity of 78.5%. The AUC, optimal cut-points, and corresponding sensitivity/specificity for each individual biomarker’s ability to discriminate between amyloid groups are presented in Table 2. This far outperformed the ability of clinical characteristics to predict amyloid status (Table S5), including age (AUC = 0.568, 95% CI 0.470–0.667), *APOE4* carrier status (AUC = 0.630, 95% CI 0.537–0.724), and age + *APOE4* carrier status (AUC = 0.674, 95% CI 0.584–0.765). An integrated model of age, *APOE4* status, and plasma AB42/40 did not significantly outperform plasma AB42/40 alone (0.847, 95% C.I. 0.781–0.913).

**Fig. (4).**
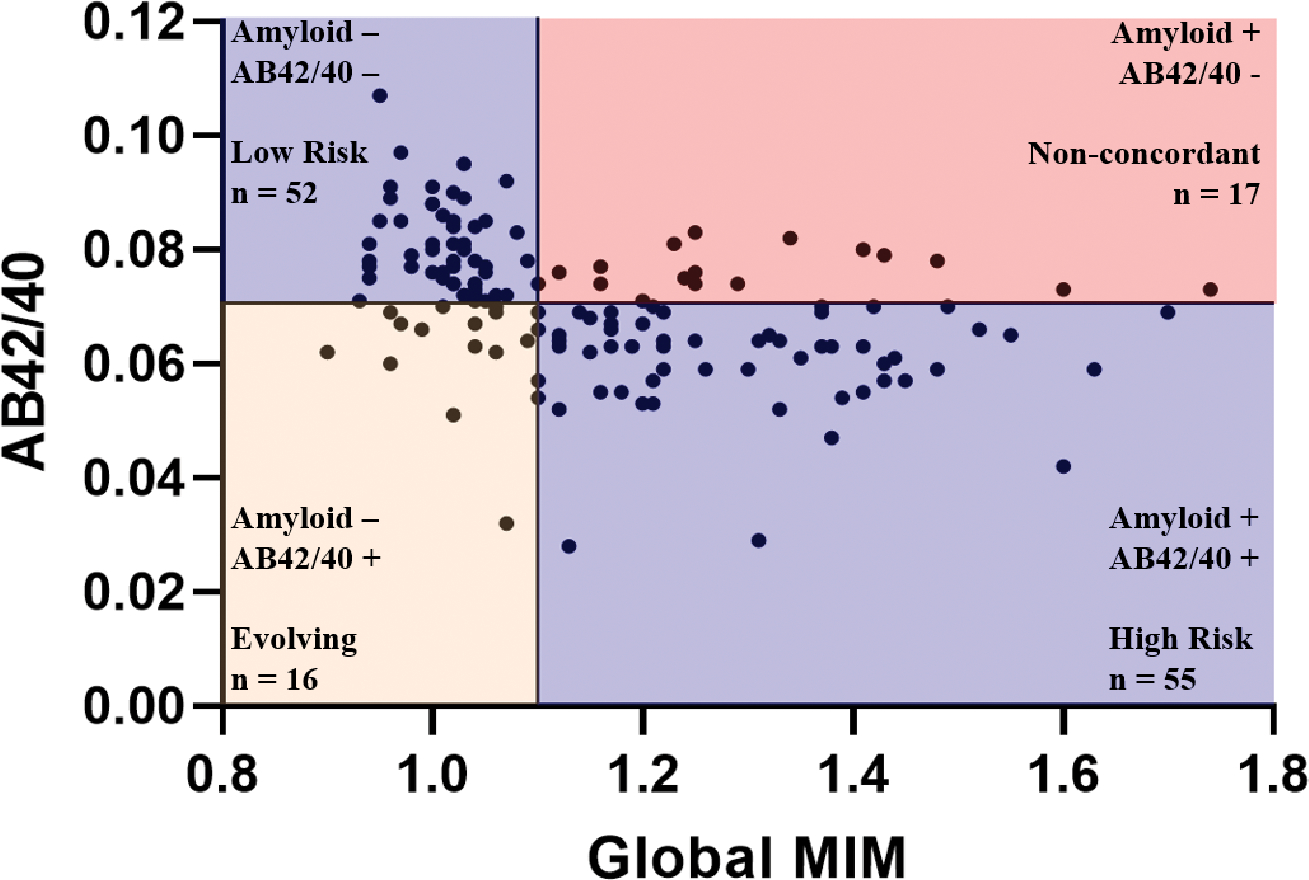
Optimal cutoff for determining elevated status in cognitively unimpaired older adults. The line plotted on the y-axis corresponds to our determined optimal cut-off for plasma AB42/40 of 0.0706. The line plotted on the x-axis corresponds to a Global MIM value of 1.1, which has been established as a cut-off for cerebral amyloid positivity 32. Sample sizes for each classification group are shown. Cerebral amyloid-negative and plasma amyloid-negative individuals were classified as “low risk”. Cerebral amyloid-positive and plasma amyloid-positive individuals were classified as “high risk”. Cerebral amyloid-positive and plasma amyloid-negative individuals were classified as “non-concordant”. The group that was cerebral amyloid negative and plasma amyloid positive, while also displaying non-concordant test results, was classified as “evolving” to distinguish them from the previous group, and based on evidence that these individuals may be at high risk of developing cerebral amyloid positivity in the future.

We visualized the relationship between plasma AB42/40 ratio and cerebral amyloid positivity in Fig. (4). Cerebral amyloid-negative and plasma amyloid-negative individuals were classified as “low risk”. Cerebral amyloid-positive and plasma amyloid-positive individuals were classified as “high risk”. Cerebral amyloid-positive and plasma amyloid-negative individuals were classified as “non-concordant”. The group that was cerebral amyloid negative and plasma amyloid positive, while also displaying non-concordant test results, was classified as “evolving” to distinguish them from the previous group, and based on evidence that these individuals may be at high risk of developing cerebral amyloid positivity in the future.

### 3.3. Plasma Biomarkers, Specifically NFL, Discriminate Cognitive Status

The mean, standard deviation, and significance of the effect of cognitively impaired status on each plasma biomarker (except GFAP, not available in the impaired cohort) are summarized in Table 1. There was no effect of cognitive status on plasma AB42 (unimpaired, 10.12 ± .2.65; impaired, 9.90 ± 3.08). However, for both plasma AB40 (unimpaired, 145.1 ± 32.1; impaired, 182.6 ± 67.1) and AB42/40 ratio (unimpaired, .0701 ± .0125; impaired, .0561 ± .0105), there existed a significant effect of cognitive status.

PTau-181 was higher in the impaired group (4.04 ± 1.97) compared to the pooled CH group (2.55 ± 1.02). However, NfL displayed the largest difference between the impaired and CH group, with an increase of 126.6% in the impaired group (30.41 ± 12.81) compared to CH (13.42 ± 5.59). The effect of cognitive status on each biomarker was not affected by the inclusion of covariates (age, sex, and *APOE4* carrier status).

NfL had the highest AUC (0.917, 95% CI 0.861–0.973) for the ROC analysis in discriminating cognitive status (Fig. 3B). Using Youden’s Index, the optimal cut-point was determined to be 20.56pg/mL, which resulted in a sensitivity of 87.5% and a specificity of 89.9%. The AUC, optimal cutpoints, and corresponding sensitivity/specificity for each individual biomarker’s ability to discriminate between impairment groups are presented in Table 2. This far outperformed the ability of clinical characteristics to predict cognitive status (Table S6), including age (AUC = 0.525, 95% CI 0.387–0.663), *APOE4* carrier status (AUC = 0.630, 95% CI 0.474–0.691), and age + *APOE4* carrier status (AUC = 0.674, 95% CI 0.484–0.733). An integrated model of age, *APOE4* carrier status, and plasma NfL did not significantly outperform plasma NfL alone (0.917, 95% C.I. 0.862–0.971).

## 4. DISCUSSION

Amyloid-PET is used to characterize amyloid pathology *in vivo* and to identify individuals at risk for AD before symptoms. Here, we set out to characterize ACD-A plasma samples for AD blood biomarkers and to further examine their ability to discriminate cerebral amyloid status and cognitive impairment status. While the plasma AB42:40 ratio differentiates CH individuals with positive amyloid-PET imaging from those without, the AUC for ROCs ranges from 0.78 [13] to 0.89 [6]. Our results reinforce this finding (AUC=0.833) and extend the utility of AB42/40 ratio to ACD-A plasma measured by the Simoa. Our results correspond with prior work showing that this ratio outperforms AB42 alone in predicting amyloid-PET status, both for CSF [32, 33] and plasma [6, 7]. In individuals with subjective cognitive decline, plasma AB42:40 measured *via* Simoa had a nearly identical accuracy to our findings for discriminating those with elevated CSF-amyloid (AUC=0.83) [34].

Other aspects of the plasma A/T/N framework are useful in discriminating both amyloid status and diagnosis status. Phospho-tau isoforms, specifically pTau-181, reflect changes in both amyloid and tau pathology in the brain [35, 36]. In fact, plasma pTau-181 may outperform CSF pTau-181 in predicting cognitive decline (MMSE) [37]. Our results suggest a modest but statistically meaningful performance of pTau-181 for discriminating both amyloid status (AUC = 0.652) and cognitive status (AUC = 0.746). Though unavailable for this study, other pTau isoforms may also be useful. For instance, plasma pTau-231 has differentiated AD from amyloid-negative, CH individuals (AUC=0.92–0.94) [38] as well as AD patients from non-AD impaired individuals (AUC = 0.93) and from amyloid-negative MCI individuals (AUC = 0.89) [38]. Plasma pTau-231 also strongly (AUC = 0.99) differentiated individuals with AD neuropathology from those without [38]. However, it is unclear if discrepancies in isoform predictive accuracy are a result of assay-specific differences or differences in the temporal role of different pTau isoforms during AD progression [39].

The development of neurodegeneration and astrocytic biomarkers is also important for improving diagnosis and monitoring. NfL is a non-specific marker of axonal injury and brain atrophy [40–42]. GFAP reflects astrocytic activation and neuroinflammation [43–45]. NfL [42] and GFAP [43] increase across the AD spectrum. Though not specific to AD, NfL and GFAP are both associated with declines in global (and subdomain) cognitive performance, and both markers correlate positively with medial temporal lobe atrophy (Spearman’s rho > 0.33). [46] While CSF GFAP is likely to reflect neuroinflammatory changes, plasma GFAP levels may reflect amyloid-specific responses of astrocytes [47]. Our results suggest that GFAP had moderate accuracy in predicting amyloid status (AUC = 0.689).

Composite biomarkers have been explored as an alternative to single biomarker predictions. Using an IP-MS approach, a combination of plasma AB42:40 ratio and plasma GFAP best predicted amyloid status overall (AUC=0.865), while a combination of AB42:40, GFAP, and pTau-181 best predicted amyloid status in the cognitively impaired subgroup (AUC=0.935). However, plasma AB42:40 alone, as measured by IP-MS, best predicted amyloid status in the CH subgroup (AUC=0.823). This finding closely resembles our findings regarding AB42/40 as a predictor of amyloid status in CH individuals, as our present analysis of the amyloid-PET data was limited to our CH sample. We did not find that a combination of biomarkers was superior to either biomarker alone for the prediction of amyloid or cognitive status (data not shown). Additionally, we did not find that the addition of age or *APOE4* carrier status into the ROC curve for either amyloid or cognitive status alters the accuracy of these plasma biomarkers. *APOE4* is the foremost genetic risk factor for sporadic AD and has roles in amyloid clearance, plaque formation, and lipid metabolism [48]. *APOE4* carriers display lower plasma AB42, AB40, and AB42:40 ratio overall [14]. It is unclear if *APOE4* status improves [46] or does not improve [11] the diagnostic accuracy of the AB42:40 ratio in predicting cerebral amyloid status.

Studies may differ in processing methodology, such as anticoagulant type, based on emphasis on different outcomes in the study design. For instance, traditional chelation-based anticoagulants, such as EDTA, decrease counts of platelets or endothelial micro-vesicles [49]. In contrast, ACD has been shown to improve outcomes for studies focused on microparticles, such as extracellular vesicles, and does not interfere with downstream protein or RNA analyses [17]. The validated use of ACD-A as an anticoagulant may prove useful for investigators with a focus on such outcomes while also quantifying plasma biomarkers relative to ADRD neuropathology.

While standardization in biomarker collection and analysis procedures for A/T/N measures is critically important, and we do not advocate replacing EDTA-based collection strategies, our results suggest that ACD-A plasma results are comparable in discriminating cerebral amyloid status. This is applicable to studies and trials that have banked plasma in this manner. Examination of the absolute values of plasma biomarker cut-offs and group averages should be done with care, as a choice of anticoagulant can affect the absolute concentration of analytes without necessarily altering their discriminative accuracy.

An important limitation is that due to a lack of amyloid PET data in the cognitively impaired group, we cannot determine if these biomarkers predict amyloid status independent of cognitive diagnosis. However, prior work examining a subset of the biomarkers presented in this work did not find an interaction between impairment and amyloid-PET status for any plasma marker [46]. It should also be stressed that our cognitively impaired group was based on clinical assessment, and clinical etiology was not confirmed neuropathologically.

Additionally, in interpreting the performance of the NfL single-plex assay, we did not carry out a complete immunoassay validation in ACD-A plasma (due to constraints on available plasma volume and resource availability). As mentioned, the extremely high performance of the NfL measure on predicting cognitive status suggests excellent performance even when using a non-validated anticoagulant. Furthermore, as the N2PB assay used to measure GFAP was performed in serum (due to the volume constraints), we had N2PB-NFL serum measurements to compare with the single-plex-NFL ACD-A measurements. While a more extensive validation is warranted, the high degree of correspondence (R2 = 0.79, Fig. S3) between the manufacturer-validated serum N2PB-NfL and unvalidated ACD-A single-plex-NFL provides evidence of the robustness of measurement. In the case of pTau-181, the assay was not validated for ACD-A plasma, and serum results were not available. The limited performance of pTau-181 indicates that while plasma amyloid and NfL may be robustly measured in ACD plasma, ptau derivates should solely be measured in manufacturer-validated mediums until more strict validation is performed.

While we highlight the novel use of ACD-A plasma as a strength in this study, this study was not designed as a superior/inferior comparison between multiple tube types. However, the comparable performance of ACD-A plasma demonstrated in this study should provide confidence to researchers leveraging newly acquired or banked ACD-A plasma samples for the quantification of such markers. Another important limitation includes the lack of diverse recruitment in this study. All but 5 individuals (2 non-elevated, 3 elevated) identified as non-Hispanic White. Given that both race and ethnicity seem to have influences on the association between fluid biomarkers and the course of cognitive decline, we hope to replicate these findings in more diverse cohorts [50].

## CONCLUSION

Our results suggest that the Aβ42/40 ratio is useful in discriminating clinician-rated elevated cerebral amyloid status and that NFL is useful for discriminating cognitive impairment status. These findings reinforce the growing body of evidence regarding the general utility of these biomarkers and extend their utility to plasma collected in a nontraditional anticoagulant.

## Supplementary Material

Supplementary Figures

## Figures and Tables

**Table 1.** Demographics and characterization of cohorts.

-	Non-Elevated (n = 65)	Elevated (n = 75)	*P*-value (Amyloid Status)	Cognitively Impaired (n = 42)	*P*-value (Impaired Status)
Age at visit (y)	71.3 (5.3)	72.2 (5.0)	0.292	73.5 (9.4)	**<0.001**
Sex (#, % female)	37 (56.9%)	49 (65.3%)	0.528	24 (57.1%)	0.618
Education (y)	16.2 (2.5)	16.3 (2.3)	0.880	15.0 (2.7)	**0.004**
APOE Carrier Status (#, % E4 carriers)	15 (24.6%)	38 (50.7%)	**<0.001**	25 (59.5%)	**0.013**
BMI	30.0 (6.3)	27.8 (5.3)	**0.025**	27.4 (6.0)	0.176
MMSE	28.7 (2.7)	29.1 (1.0)	0.285	22.8 (5.2)	**<0.001**
AB42	11.49 (2.36)	8.93 (2.30)	<.001	9.90 (3.08)	**0.649**
AB40	150.3 (33.4)	140.5 (30.5)	0.074	182.6 (67.1)	**<0.001**
AB42/40	0.0774 (.0099)	0.0638 (.0110)	<0.001	.0561 (.0105)	**<0.001**
pTau181	2.25 (0.84)	2.80 (1.09)	0.002	4.04 (1.97)	**<0.001**
NFL	14.05 (6.15)	12.88 (5.03)	0.221	30.41 (12.81)	**<0.001**
GFAP (serum)	200.7 (131.7)	255.2 (115.1)	0.013	-*	-*
Global MIM	1.015 (.0479)	1.289 (.1597)	<0.001	-*	-*

**Note:** Demographic characteristics of cognitively unimpaired and impaired individuals included in the study. Bolded p-values indicate significance *p*<0.05.

**Abbreviations:** APOE, apolipoprotein E; BMI, body mass index; MMSE, mini-mental status exam; AB, amyloid beta; pTau, phosphorylated tau; NFL, neurofilament-light; GFAP, glial fibrillary astrocytic protein. P-values are reported for the differences between the non-elevated and elevated groups (amyloid status) and for differences between the pooled cognitively unimpaired and impaired groups (impaired status). Bolded *p*-values indicate significance after appropriate multiple comparisons correction.

* Amyloid PET and plasma GFAP were not available for the cognitively impaired cohort.

**Table 2.** Diagnostic accuracy of plasma biomarkers to detect elevated amyloid status and cognitively impaired status.

-	-	Youden's Cut Point	AUC (95% CI)	Sensitivity (%)	Specificity (%)
Elevated amyloid status	AB42:40 Ratio	0.0706	0.833 (.767-.899)	76.0	78.5
	GFAP	161.0	0.689 (.596-.782)	85.5	48.3
	pTau181	1.866	0.652 (.557-.747)	82.9	40.4
	NFL	10.59	0.457 (.359-.554)	68.9	37.5
Cognitively impaired status	NFL	20.56	0.917 (.861-.973)	87.5	89.9
	AB42/40	0.0625	0.829 (.762-.896)	78.6	77.9
	pTau181	2.97	0.763 (.683-.843)	69.1	73.2

**Note:** Listed are the sensitivity and specificity of each biomarker for discriminating cerebral amyloid status and cognitive impairment, evaluated at a cut-point determined using the maximum Youden’s Index. This index equally weighs the importance of sensitivity and specificity.

**Abbreviations:** AB, amyloid beta; GFAP, glial fibrillary acidic protein; pTau, phosphotau; NFL, neurofilament-light.

## Data Availability

The data and supportive information are available within the article.

## LIST OF ABBREVIATIONS

AD: Alzheimer’s Disease
ACD: Acid Citrate Dextrose
AUC: Area Under the Curve
NfL: Neurofilament Light
GFAP: Glial Fibrillary Acidic Protein
SUVR: Standard Uptake Value Ratio
APOE: Apolipoprotein E
CSF: Cerebrospinal Fluid
PET: Positron Emission Tomography
MMSE: Mini-mental State Examination
MIM: Medical Image Merge
