# Supplementary material for: Blood Biomarkers Discriminate Cerebral Amyloid Status and Cognitive Diagnosis when Collected with ACD-A Anticoagulant
Source: Curr Alzheimer Res. Author manuscript; Available in PMC 2024 Jan 17. (PMC10792989; doi:10.2174/0115672050271523231111192725)

Supplementary Materials

Blood Biomarkers Discriminate Cerebral Amyloid Status and Cognitive Diagnosis when Collected with ACD-A Anticoagulant

Zachary D. Green[1], Paul J. Kueck[1], Casey S. John[1], Jeffrey M. Burns[1,2] and Jill K. Morris[1,2,*]

[1]*Alzheimer's Disease Research Center, University of Kansas, Kansas City, KS, 66160, United States;* [2]*Department of Neurology, University of Kansas Medical Center, Kansas City, KS, 66160, United States*

Supplementary Figures/Tables

Supplementary Table 1 – *Partial validation results for N3PA kit in ACD plasma*

	LLOQ (pg/mL)	Parallelism (%)	Repeatability		Intermediate Precision	
			%CVr Low	%CVr High	%CV.Rw Low	%CV.Rw High
Ab42	0.17	103	4.1	2.9	5.1	3.7
Ab40	1.54	88	4.8	3.3	6.9	4.9
Tau	0.32	106	6.6	5.6	6.6	7.0

Supplementary Table 2 – Means and standard deviations of amyloid-PET ROIs

	**All Amyloid Negative (n=65)**	**APEX Elevated (n=75)**
Anterior Cingulate	1.0749 (.08172)	1.3843 (.19461)
Inferior Medial Frontal	0.9466 (.06145)	1.2136 (.17109)
Lateral Temporal	1.0385 (.06741)	1.2925 (.16349)
Posterior Cingulate	1.0158 (.05905)	1.2624 (.16028)
Precuneus	1.0415 (.0682)	1.3828 (.20693)
Superior Parietal	0.9709 (.08058)	1.1963 (.17355)

Supplementary Table 3 – Correlation between biomarkers in full sample, and in the cognitively impaired group alone

ND AND AD

	AB_42	AB_40	AB42/40	pTau181	NFL
AB_42	1	0.690	0.400	-0.016	0.251
AB_40		1	-0.351	0.350	0.639
AB42/40			1	-0.443	-0.399
pTau181				1	0.495
NFL					1

AD Only

	AB_42	AB_40	AB42/40	pTau181	NFL
AB_42	1	0.830	0.021	0.041	0.321
AB_40		1	-0.512	0.241	0.665
AB42/40			1	-0.372	-0.574
pTau181				1	0.389
NFL					1

-1
-0.8
-0.6
-0.4
-0.2
0
0.2
0.4
0.6
0.8
1

 © XXXX Bentham Science Publishers

Supplementary Table 4 – Correlations between plasma biomarkers and individual amyloid-PET ROIs

ND Only

	AB_42	AB_40	AB42/40	pTau181	NFL	GFAP
AntCing_MIM	-0.378	-0.137	-0.387	0.271	-0.082	0.259
InfMedFront_MIM	-0.381	-0.148	-0.379	0.274	-0.104	0.242
LatTemp_MIM	-0.431	-0.211	-0.386	0.277	-0.069	0.300
PostCing_MIM	-0.384	-0.162	-0.367	0.307	-0.006	0.317
Precuneus_MIM	-0.392	-0.153	-0.384	0.300	-0.036	0.301
SupParietal_MIM	-0.321	-0.100	-0.351	0.253	-0.049	0.328
Global_MIM	-0.407	-0.161	-0.402	0.300	-0.062	0.310

-1
-0.8
-0.6
-0.4
-0.2
0
0.2
0.4
0.6
0.8
1

Supplementary Table 5 - *Receiver Operating Characteristic performance for clinical characteristics and combined AB42/40 + clinical characteristics for predicting cerebral amyloid status*

	AUC (95% CI)	Sensitivity (%)	Specificity (%)
Age	0.568 (.470-.667)	61.3	57.4
APOE	0.630 (.537-.724)	50.7	75.4
Age + APOE	0.674 (.584-.765)	80.0	49.2
Age + APOE + AB42/40	0.847 (.781-.913)	80	78.7

Supplementary Table 6 - *Receiver Operating Characteristic performance for clinical characteristics and combined NFL + clinical characteristics for predicting cognitive impairment*

	AUC (95% CI)	Sensitivity (%)	Specificity (%)
Age	0.525 (.387-.663)	48.5	79.1
APOE	0.583 (.474-.691)	50	42.4
Age + APOE	0.609 (.484-.733)	39.4	87.8
Age + APOE + NFL	0.917 (.862-.971)	78.8	89.9

Supplementary Figure 1 – ***Biomarker comparison between ACD and EDTA plasma from a developmental cohort***

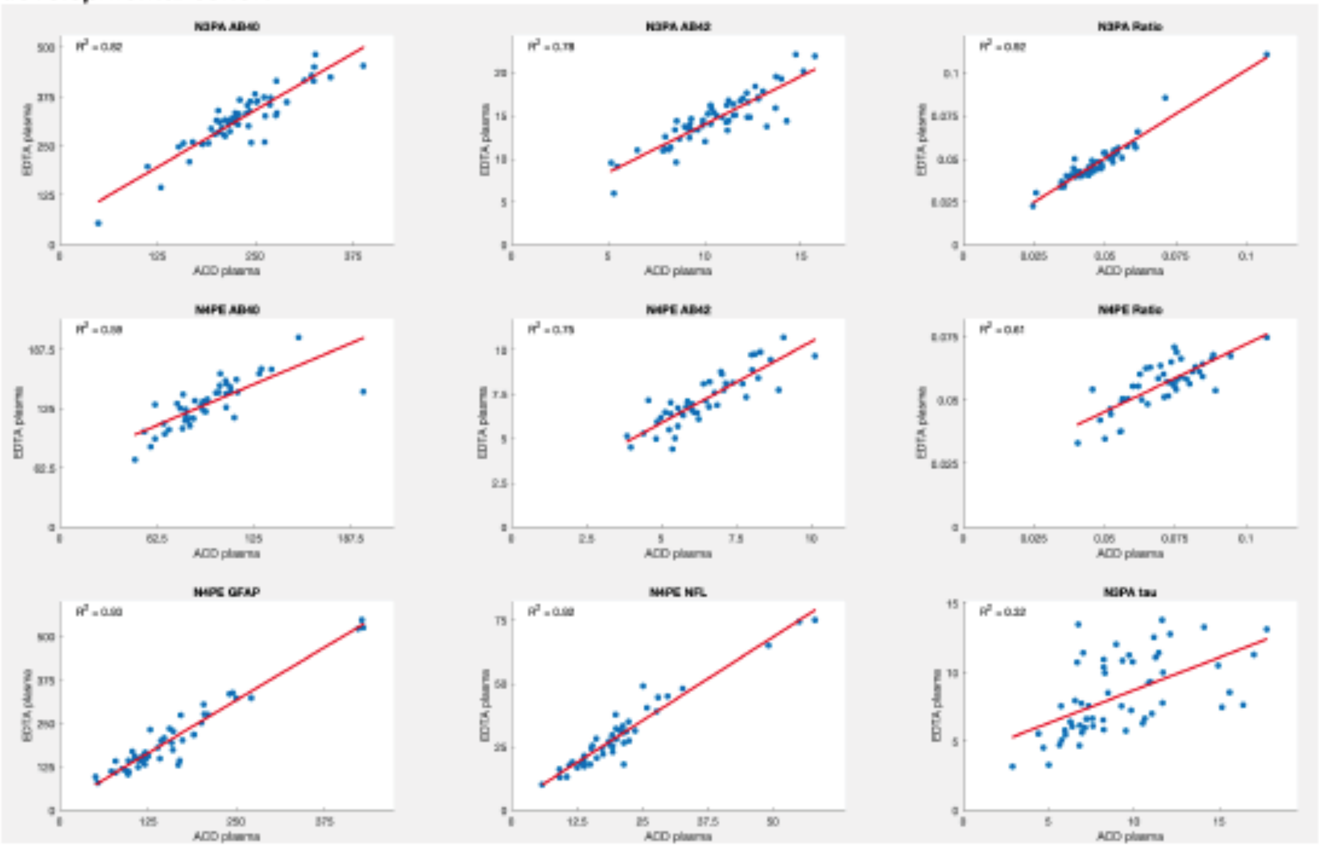

We leveraged existing biospecimens from a separate developmental cohort (cite NIS?). This developmental cohort did not have any amyloid-PET data, but was instead used to evaluate how robust the measurement of the plasma biomarkers were to kit type and tube type. Specifically, we validated individual differences in AB42, AB40, and AB42/40 ratio by measuring them in both ACD and EDTA plasma, using both the Neuro 3-Plex (N3PA) and Neuro 4-Plex (N4PE) kits offered.

Supplementary Figure 1 details the associations between each tube type when measured using the same kit. As the N3PA kit appeared to show less measurement variance between ACD and EDTA plasma (R^2 = 0.92, 95% CI [0.87, 0.95]) when compared to N4PE (R^2 = 0.61, 95% CI [0.40, 0.76]) we opted to use former to test for amyloid biomarkers in the present study cohort (Supplementary Figure 1, top right and far right panels).

Supplementary Figure 2 – ***Biomarker comparison between N3PA and N4PE kits in EDTA/ACD plasma from a developmental cohort***

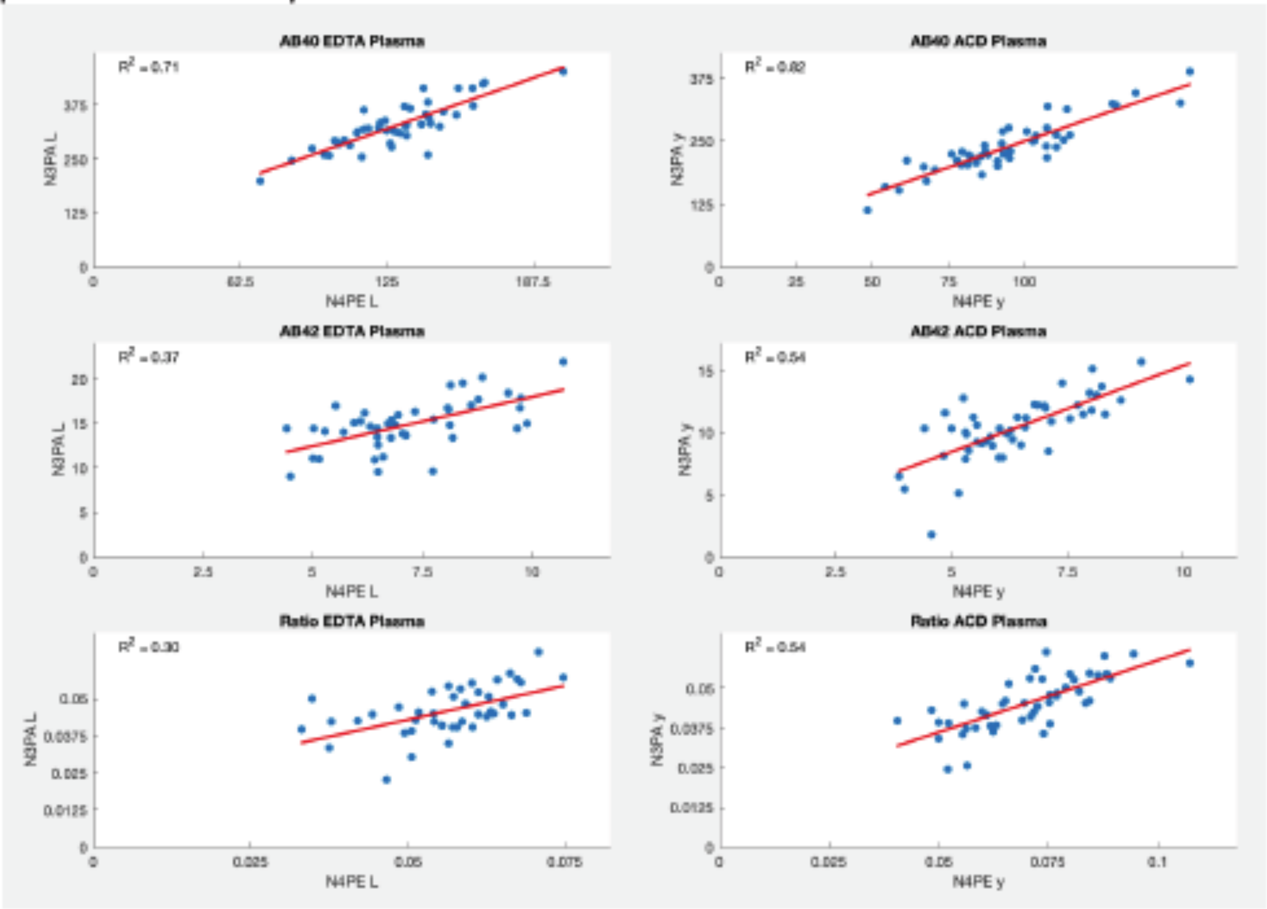

In Supplementary Figure 2, we show that coherence between the two amyloid-measuring kits is not high regardless of anticoagulant type. For instance, in EDTA plasma, correlations were moderate for AB42 (R^2 = 0.37) and AB42/40 ratio (R^2 = 0.30), and moderate-high for AB40 (R^2 = 0.71).

Interestingly, while still showing only a moderate correlation, the correlation between kits was significantly higher in ACD plasma for AB42/40 ratio (R^2 = 0.54, 95% CI [0.32, 0.71]). This further informed our decision to measure the plasma amyloid biomarkers using the N3PA kit in the main study cohort. Correlations between kits were higher in ACD plasma, but not significantly so, for AB42 (R^2 = 0.54, 95% CI [0.33, 0.71]) and AB40 (R^2 = 0.71, 95% CI [0.53, 0.83]).

Supplementary Figure 3 – *Scatterplot of NFL collected via single-plex in ACD plasma against NFL collected via N2PB in serum (main study cohort, cognitively unimpaired only)*

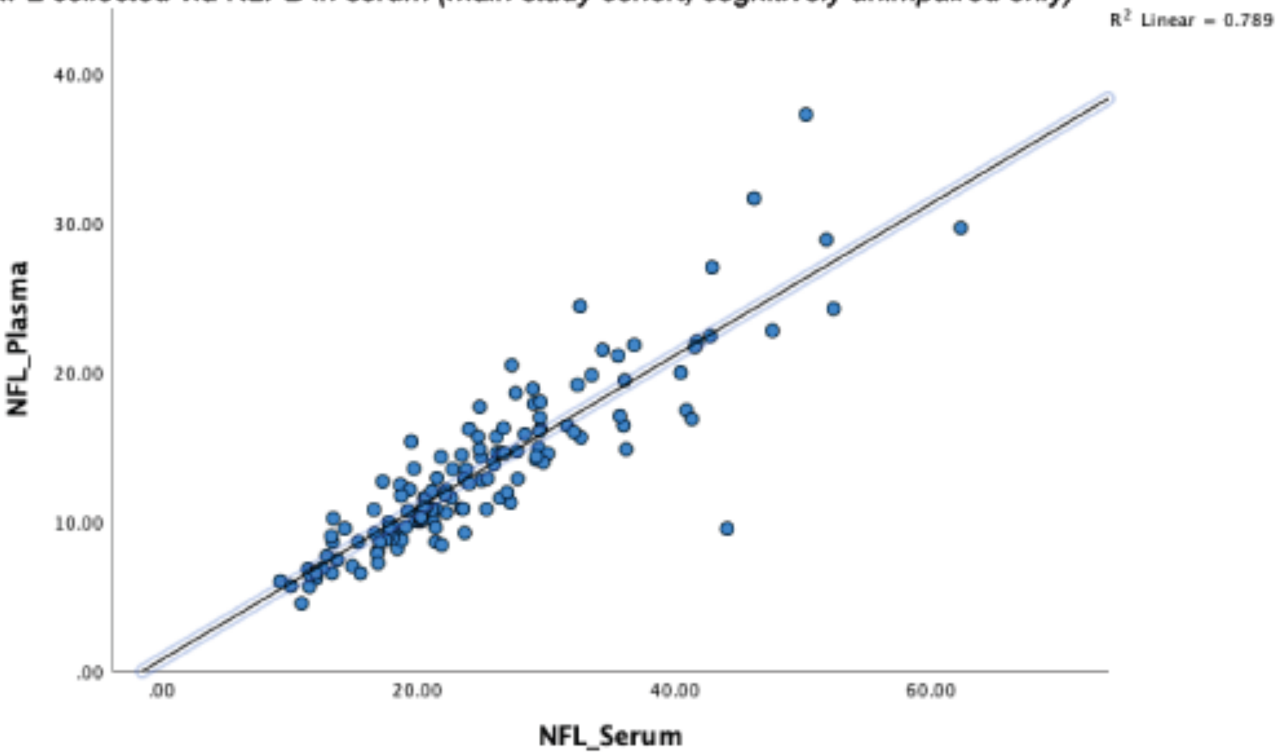

Supplement: Supplementary file 1 [file NIHMS1957430-supplement-Supplementary_Figures.pdf]
